# Sex-specific brain morphological and network differences in patients showing Parkinson's disease with and without possible rapid eye movement sleep behavior disorder

**DOI:** 10.3389/fneur.2025.1561555

**Published:** 2025-04-22

**Authors:** Yang Liu, Pengfei Zhang, Hao Li, Liang Zhou, Jingqi Jiang, Yanli Jiang, Kai Ai, Guangyao Liu, Jing Zhang

**Affiliations:** ^1^The Second Hospital & Clinical Medical School, Lanzhou University, Lanzhou, China; ^2^Gansu Province Clinical Research Center for Functional and Molecular Imaging, Lanzhou, China; ^3^Gansu Medical MRI Equipment Application Industry Technology Center, Lanzhou, China; ^4^Department of Clinical and Technical Support, Philips Healthcare, Xi'an, China

**Keywords:** Parkinson's disease, sex differences, rapid eye movement sleep behavior disorder, morphological brain network, cortical surface

## Abstract

**Background:**

Sex is a crucial determinant in the clinical manifestations of diseases. However, previous studies have not clarified whether altered brain morphology shows sex-specific patterns in patients with Parkinson's disease (PD) with or without possible rapid eye movement sleep behavior disorder (RBD). This study aimed to investigate sex-specific differences in the patterns of morphological changes among different subgroups of PD.

**Methods:**

High-resolution T1-weighted magnetic resonance imaging and clinical scale data were collected from 278 participants in the Parkinson's disease Progression Marker Initiative database: 93 patients with PD-pRBD (60 males, 33 females), 114 patients showing PD without RBD (PDnon-pRBD group; 68 males, 46 females), and 71 healthy controls (HCs; 44 males, 17 females). The Computational Anatomy Toolbox (CAT) 12 was utilized to collect data on gray matter volume (GMV) and cortical morphological metrics. Subsequently, individual-level morphological similarity networks were constructed on the basis of these cortical metrics. Finally, the topological properties of the network were analyzed using graph theoretical methods.

**Results:**

In the PD-pRBD group, the GMV in the frontal and temporal lobes of males was lower than that of females. In contrast, the gyrification index (GI) of the frontal lobe in males was lower than that in females within the PDnon-pRBD group. Network analyses based on graph theory revealed that male PD-pRBD patients showed lower network information integration than female patients, particularly in terms of the global properties of fractal dimension (FD) networks. Moreover, in the PD-pRBD group, male patients showed a strong correlation between morphological network metrics and cognitive performance, as measured by the Hopkins Verbal Learning Test-Revised (HVLT*-*R) memory scores.

**Conclusion:**

The presence of more significant sex-related differences in brain morphological changes in the PD-pRBD group in comparison with the PDnon-pRBD group highlights the importance of considering sex-related differences in the diagnosis and management of patients with PD-pRBD.

## Introduction

Parkinson's disease (PD) is one of the most prevalent neurodegenerative disorders in older adults, with its incidence surpassed only by that of Alzheimer's disease (1, 2). Emerging studies have indicated significant associations between sex and the incidence as well as clinical severity of various complex diseases, particularly neurodegenerative disorders (3, 4). Specifically, studies have indicated that the prevalence of PD is generally higher among males than females (5), with sex-related differences prominently observed in clinical presentation, disease progression, treatment response, and pathological features (6, 7). Therefore, a deeper understanding of the sex-specific neuropathological mechanisms in PD is crucial for advancing the development of individualized therapeutic strategies.

The typical clinical manifestations of PD are primarily movement disorders; however, highly heterogeneous non-motor symptoms can also significantly influence patients' quality of life, of which rapid eye movement sleep behavior disorder (RBD) is one of the most common non-motor symptoms of PD (8). RBD is typically closely associated with synucleinopathies which is considered a prodromal stage of neurodegenerative diseases and potentially occurring years or even decades before the typical clinical symptoms of these diseases emerge. For these patients, RBD not only further worsens sleep quality and increases the risk of nocturnal trauma but may also serve as an important predictor of dementia risk (9). Previous studies have indicated that patients with PD and RBD (PD-pRBD) tend to exhibit more severe disease manifestations and experience faster disease progression (10, 11). Bjornara et al. (12) reported that the overall prevalence of RBD in patients with PD was 43% in males and 31% in females. They also observed significant differences in symptom presentation and cognitive performance between sexes among patients with PD-pRBD. Female patients with PD-pRBD exhibited more symptoms of sleep disturbance, whereas male patients with PD-pRBD displayed more violent behaviors (13, 14). Furthermore, studies have suggested that male patients with PD-pRBD exhibit significantly worse cognitive performance than female patients (5). However, this difference is not significant in patients with PD without RBD (15, 16). These findings suggest that RBD may be a key factor in sex-specific neural alterations in PD patients.

Clinical symptoms are often closely linked to structural and functional alterations in the central nervous system, with male and female patients potentially showing distinct patterns of brain changes. Magnetic resonance imaging (MRI), as a non-invasive modality, enables detailed analysis of brain structure and function and holds significant promise for investigating the central mechanisms underlying PD (17–19). MR-based morphological studies have confirmed the presence of sex-related structural differences in the brains of patients with PD. In comparison with females, males exhibit a wide range of structural abnormalities across multiple cortical regions, including the frontal, parietal, and temporal lobes (20). Furthermore, male PD patients with RBD were found to show more subcortical structural atrophy than female PD patients, and this sex-specific difference in cortical atrophy was more pronounced than that in PD patients without RBD (5). However, most current studies on patients with PD-pRBD have primarily focused on gray matter volumes, with fewer studies addressing cortical morphology. Moreover, with advances in the understanding of neural networks, studies have demonstrated that brain morphology shows diverse and synergistic patterns of change at different stages of development (21–23), closely correlating with patients' clinical symptoms. These factors highlight the urgent need for a systematic and comprehensive analysis of the interconnections between brain regions at the network level, building on existing studies of brain morphology.

Therefore, we aimed to perform brain-based cortical morphological studies to further explore these covariation and gain insights into sex-specific morphological features in PD patients with or without RBD. These findings will potentially help elucidate the pathophysiological mechanisms of PD more comprehensively and further explain the changes in patient behavior.

## Materials and methods

### Participants

T1-weighted imaging and clinical scale data from 340 participants were sourced from the Parkinson's Disease Progression Marker Initiative (PPMI) (24) (PPMI, http://www.ppmi-info.org). A total of 62 participants with images of inadequate quality (Computational Anatomy Toolbox image quality ratings <75%) (25) were excluded, resulting in a final cohort of 278 participants. Patients with PD-pRBD were identified on the basis of a cu*t-*off score of 5 points on the RBD Screening Questionnaire (RBDSQ) (26). The final cohort consisted of six groups, including 93 patients with PD-pRBD (60 males, 33 females), 114 patients showing PD without RBD (PDnon-pRBD group; 68 males, 46 females), and 71 healthy controls (HCs; 44 males, 17 females).

The inclusion criteria for patients with PD were as follows: (1) presence of asymmetric resting tremors, asymmetric bradykinesia, or both; (2) completion of comprehensive clinical evaluations; (3) absence of systemic diseases that could affect neurological assessment; and (4) availability of T1-weighted images.

The inclusion criteria for HCs were as follows: (1) availability of T1-weighted images; (2) completion of clinical assessment; and (3) absence of systemic diseases that could affect neurological assessments.

Exclusion criteria for all participants were as follows: (1) a diagnosis of dementia; (2) history of psychiatric or neurological disorders; (3) organic cranial brain lesions or prior cranial brain surgery; (4) a firs*t-*degree family member with PD; and (5) MRI scans with suboptimal quality.

The institutional review boards of all participating centers approved the PPMI study, and written informed consent was obtained from all participants by the center investigators in compliance with the Declaration of Helsinki (please refer to Supplementary material for detailed information).

### Clinical and neuropsychological assessments

The patients underwent a comprehensive clinical assessment, including evaluation of PD symptoms using the Movement Disorder Society Unified Parkinson's Disease Rating Scale (MDS-UPDRS). Motor symptoms in PD were assessed using the motor portion of the MDS-UPDRS (Part III) (27). Tremor scores were derived from items 15–18 of the UPDRS-III, and rigidity was assessed using item 3. Disease severity was measured by the Hoehn and Yahr scale (H&Y) (28). Overall cognitive function was evaluated using the Montreal Cognitive Assessment (MoCA) (29), and depressive symptoms were assessed with the 15-item Geriatric Depression Scale (GDS-15) (30). Possible RBD states and symptoms were assessed using the RBDSQ (27). The State-Trait Anxiety Inventory (STAI-S) is recognized as a valid tool for assessing anxiety symptoms in individuals with early PD (31). Additionally, all participants underwent neuropsychological testing using the Letter-Number Sequencing (LNS) for assessing executive functioning and working memory (32), the Benton Judgment of Line Orientation Short Form (BJLOT) for evaluating visuospatial functioning (33), and the Hopkins Verbal Learning Test-Revised (HVLT*-*R) for investigating memory (16). The Questionnaire for Impulsive-Compulsive Disorders in Parkinson's Disease (QUIP) was used to evaluate the degree of impulse control disorder in patients (34).

### Magnetic resonance imaging acquisition

T1-weighted MRI scans were acquired using a 3.0T scanner (Trio™ or Verio™ system, Siemens Healthcare) with a magnetization-prepared rapid gradient echo imaging (MPRAGE) sequence. The MRI parameters were as follows: repetition time = 1.9–3 ms; echo time = 2,300 ms; slice thickness = 1–1.2 mm; voxel size = 1 × 1 × (1–1.2) mm; matrix size = 240 × 256 minimum.

### Preprocessing

In this study, the SPM12-based CAT12 extension package was used for processing and analyzing VBM- and SBM-based data using the 2018a version of MATLAB software (MathWorks, Natick, Massachusetts, USA). Initially, the 3D-T1WI images of each participant underwent a rigorous quality check to exclude individuals with excessive head movement and artifacts that could interfere with the image segmentation and normalization steps. For voxel-based morphometry (VBM) analysis, the images underwent bias field correction, de-cranialization, and segmentation into gray matter (GM), white matter (WM), and cerebrospinal fluid (CSF). Subsequently, the images were transformed to the MNI standard space using the DARTEL algorithm, and then resampled to a resolution of 1.5 × 1.5 × 1.5 mm3. Finally, the images were smoothed using an 8-mm full-width half-maximum (FWHM) Gaussian filter. For the surface-based morphometry (SBM) analysis, the cortical morphological indices of fractal dimension (FD), gyrification index (GI), sulcal depth (SD), and cortical thickness (CT) were extracted on the basis of the standardized procedure of CAT12. A 12-mm FWHM Gaussian smoothing kernel was applied to compute CT, while a 20-mm FWHM Gaussian smoothing kernel was used for the remaining metrics, in accordance with the recommendations of the official CAT12 manual (35).

To construct a morphological similarity network at the individual level, this study segmented the entire brain cortex into 68 bilateral regions based on the DK40 template (36) (a2005s template). Kernel density estimation was employed to derive the probability density function of cortical metrics for each brain region. Statistical similarities between different brain regions were evaluated by calculating the Kullback–Leibler divergence (KLD), which was then transformed to generate KLD-based similarity metrics (KLDs) (37). This metric encapsulates the morphological similarity relationships between brain regions, thereby forming a morphological similarity network.

We utilized a sparsity threshold, S (calculated as the ratio of the actual number of edges to the maximum possible edges in a network), to transform each matrix *C*_ij_ = [*c*_ij_] into both a binary and weighted network by employing a subjec*t-*specific KLS threshold.


$$A_{ij}=[a_{ij}]=\{\begin{array}{cc} 1, & if\;c_{ij}>KLS_{thr}\\ 0, & otherwise \end{array}$$

Weighted Network:


$$W_{ij}=[w_{ij}]=\{\begin{array}{cc} c_{ij}, & if\;c_{ij}>KLS_{thr}\\ 0, & otherwise \end{array}$$

This approach to thresholding guarantees that the resultant networks maintain an identical number of nodes and edges across all participants. We selected a sparsity range of 0.063–0.4 (with intervals of 0.01), consistent with previous studies (38, 39), to ensure that the resulting networks exhibit sparsity characteristics and are suitable for small-world properties.

Network graph theory analysis was conducted using the GRETNA software (40) to determine the topological properties of each network. The area under the curve (AUC) value of each attribute across these thresholds was computed as its composite representation. Subsequently, global attributes reflecting network integration (*Eg, Lp*, λ), network separation (*Eloc, Cp*, γ), and small-worldliness (σ), as well as node attributes (*Ne, Dc, Bc*), were calculated (41–44).

### Statistical analysis

Demographic data were statistically analyzed using SPSS version 26.0 software (IBM, Armonk, NY, USA). Categorical variables were presented as proportions and evaluated using chi-square tests. The Shapiro–Wilk test for normality was performed on all data. Normally distributed continuous variables were expressed as mean ± standard deviation and evaluated using two-sample *t-*tests. On the other hand, continuous variables with skewed distributions were reported as median and interquartile range (P25, P75) and compared using Mann-Whitney U and Kruskal-Wallis tests; a *P-*value <0.05 was considered statistically significant.

In this study, imaging data were statistically analyzed using the CAT12/SPM12 statistical module, with two-sample *t-*tests applied to each morphometric measure after accounting for age, educational level, and levodopa equivalent daily dose (LEDD) as covariates. For VBM analyses, the total intracranial volume (TIV) was additionally included as a covariate (39, 40). Both VBM and SBM analyses were corrected for multiple comparisons using the Family-Wise Error (FWE) method, with *P* < 0.001 and *P* < 0.05 indicating statistical significance at the voxel/vertex and clump levels, respectively. Intragroup differences in graphological metrics were corrected for nodal metrics using a non-parametric permutation test (10,000 permutations) with false discovery rate (FDR) correction. Age, LEDD, and educational level were considered as covariates. Furthermore, mean morphological metrics and topological attributes of brain regions with within-group differences were correlated with clinical variables using Pearson correlation analysis to explore potential relationships. Correlations were performed using SPSS 26.0, and statistical significance was set at *P* < 0.05.

## Results

### Clinical and demographic characteristics: sex comparisons

Demographic and clinical information for all participants is summarized in Table 1. No significant differences were observed between males and females in terms of age and educational level across the PD-pRBD, PDnon-pRBD, and HC groups. In the PD-pRBD group, significant differences (*P* < 0.05) were noted in overall cognition (MoCA score). Specifically, female participants obtained significantly higher Delayed Recall scores than male participants in the HVLT*-*R (*P* < 0.001). Female participants in both the PDnon-pRBD and HC groups obtained lower scores than male participants on the BJLOT (*P* < 0.05). Statistical analysis of baseline data among the three groups was performed. For detailed information, please refer to Supplementary Table S2.

**Table 1:** Demographic and clinical characteristics[4mm]Q20 of HC, PDnon-pRBD, and PD-pRBD.

	**HC (M/F 44/27)**	**PDnon-pRBD (M/F 68/46)**	**PD-pRBD (M/F 60/33)**
**Age, years**			
M	62.52 ± 6.75	62.23 ± 10.67	64.05 ± 8.74
F	59.78 ± 6.51	60.24 ± 8.18	62.15 ± 10.24
Within-group sex effect, test stat (*P-*value)	1.686 (0.096)	1.07 (0.287)	−0.942 (0.349)
**Education, years**			
M	17 (16–18)	16 (14–18)	16 (14–18)
F	16 (12–18)	16 (12.5–18)	16 (12.5–18)
Within-group sex effect, test stat (*P-*value)	−1.922 (0.055)	−0.787 (0.432)	0.975 (0.329)
**TIV (cm** ^3^ **)**			
M	1,491.15 (1,440.41–1,590.52)	1,589.09 (1,503.38–1,679.34)	1,567.84 (1,506.27–1,638.25)
F	1,353.06 (1,280.70–1,449.13)	1,383.96 (1,330.90–1,450.77)	1,396.64 (1,292.96–1,458.37)
Within-group sex effect, test stat (*P-*value)	−4.382 (<0.001^**^)	−6.989 (<0.001^**^)	−6.359 (<0.001^**^)
**LEDD**			
M	NA	0 (0–0)	0 (0–0)
F	NA	0 (0–0)	0 (0–335)
Within-group sex effect, test stat (*P-*value)	NA	−0.314 (0.753)	−1.995 (0.046^*^)
**UPDRS-III**			
M	NA	20.00 (15.00–28.50	25.50 (16.00–34.00)
F	NA	20.00 (13.75–27.25)	23.00 (16.00–31.00)
Within-group sex effect, test stat (*P-*value)	NA	−0.451 (0.652)	−0.462 (0.644)
**HY**			
M	NA	2 (1–2)	2 (1–2)
F	NA	2 (1–2)	2 (2–2)
Within-group sex effect, test stat (*P-*value)	NA	−1.405 (0.16)	−1.026 (0.305)
**Tremor**			
M	NA	4 (2–6)	4 (2–7)
F	NA	5 (2–7)	3 (1.5–6)
Within-group sex effect, test stat (*P-*value)	NA	−1.187 (0.235)	−0.897 (0.370)
**Rigidity**			
M	NA	3.5 (2–6)	5 (2–8)
F	NA	3 (1.75–4.25)	4 (2.5–6)
Within-group sex effect, test stat (*P-*value)	NA	1.487 (0.137)	0.929 (0.353)
**UPDRS-II**			
M	NA	4 (2–8)	6 (3–11)
F	NA	3 (2–6)	6 (3.5–8.5)
Within-group sex effect, test stat (*P-*value)	NA	–1.888 (0.059)	−0.338 (0.735)
**UPDRS-I**			
M	NA	1 (0–2)	1 (0–2)
F	NA	0 (0–2)	2 (0.5–4)
Within-group sex effect, test stat (*P-*value)	NA	−0.498 (0.619)	−1.792 (0.073)
**GDS**			
M	5 (5–6)	5 (5–6)	5 (4–6)
F	5 (5–6)	5 (4.75–5.25)	5 (4–6)
Within-group sex effect, test stat (*P-*value)	−0.521 (0.602)	−1.915 (0.056)	−0.530 (0.596)
**STAI-S**			
M	48.5 (44.25–50.75)	47.34 ± 5.05	48 (44–50)
F	49 (47–50)	47.70 ± 4.43	47 (44.5–50.5)
Within-group sex effect, test stat (*P-*value)	−0.959 (0.338)	−0.389 (0.698)	−0.475 (0.635)
**LNS**			
M	11 (9–12)	11 (9–12)	11 (9–12.75)
F	11 (10–13)	11 (9.75–12.25)	11 (9–12)
Within-group sex effect, test stat (*P-*value)	−0.862 (0.389)	−0.999 (0.318)	−0.239 (0.811)
**BJLOT**			
M	14 (13–15)	14 (13–15)	13 (12–15)
F	13 (12–14)	13 (10.75–15)	12 (10–14)
Within-group sex effect, test stat (*P-*value)	−3.011 (0.003^**^)	−2.303 (0.021^*^)	1.943 (0.052)
**MoCA**			
M	28 (27–29)	28 (26–29)	27 (25.25–28)
F	28 (27–30)	28.5 (27–29.25)	29 (26–29)
Within-group sex effect, test stat (*P-*value)	−0.018 (0.985)	−0.929 (0.353)	−2.577 (0.010^*^)
**Delayed recall of HVLT** ***-*** **R**			
M	9 (7–11)	9 (7–11)	7 (5.25–9)
F	10 (9–11)	10 (8–11)	10 (9–11)
Within-group sex effect, test stat (*P-*value)	−1.049 (0.294)	−1.556 (0.120)	−4.684 (<0.001^**^)

Data are shown as mean ± standard deviation or median (P25, P75). P-values of intragroup comparisons were obtained using t-tests and non-parametric tests, with significance set at P <0.05. ^*^Denotes P <0.05 and ^**^ denotes P <0.01. UPDRS, Unified Parkinson's Disease Rating Scale; HY, Hoehn and Yahr stage; GDS, Geriatric Depression Scale; STAI-S, State-Trait Anxiety Inventory–State; LNS, Letter-Number Sequencing; BJLOT, Benton Judgement of Line Orientation; MoCA, Montreal Cognitive Assessment; HVLT-R, Hopkins Verbal Learning Test-Revised; LEDD, Levodopa equivalent daily dose; TIV, total intracranial volume.

### Voxel-based morphometry

**Figure 1:**
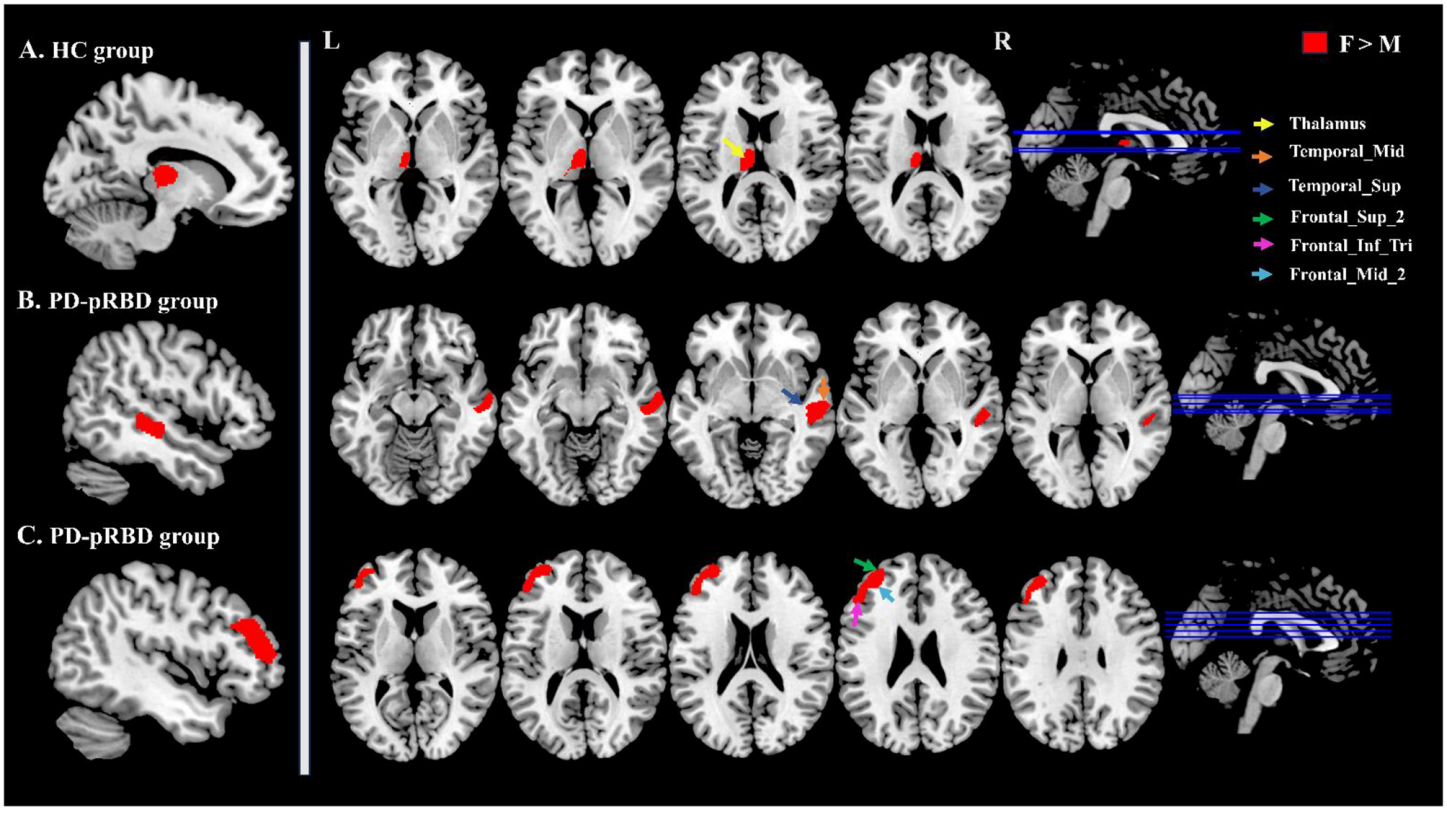
Brain regions showing intragroup differences between males and females analyzed by VBM. **(A)** Shows the findings for the HC group, and **(B, C)** show the findings for the PD-pRBD groups. The red area represents a higher GMV in females than in males. M, male; F, female.

In the PD-pRBD group, male patients exhibited smaller GMV than female patients in the right Temporal_Sup and Temporal_Mid and in the left Frontal_Sup_2, Frontal_Mid_2, and Frontal_Inf_Tri. In the PDnon-pRBD group, no significant differences in GMV were observed between male and female patients. In the HC group, male participants demonstrated a smaller GMV in the left thalamus than female participants (Figure 1).

### Surface-based morphometry

**Figure 2:**
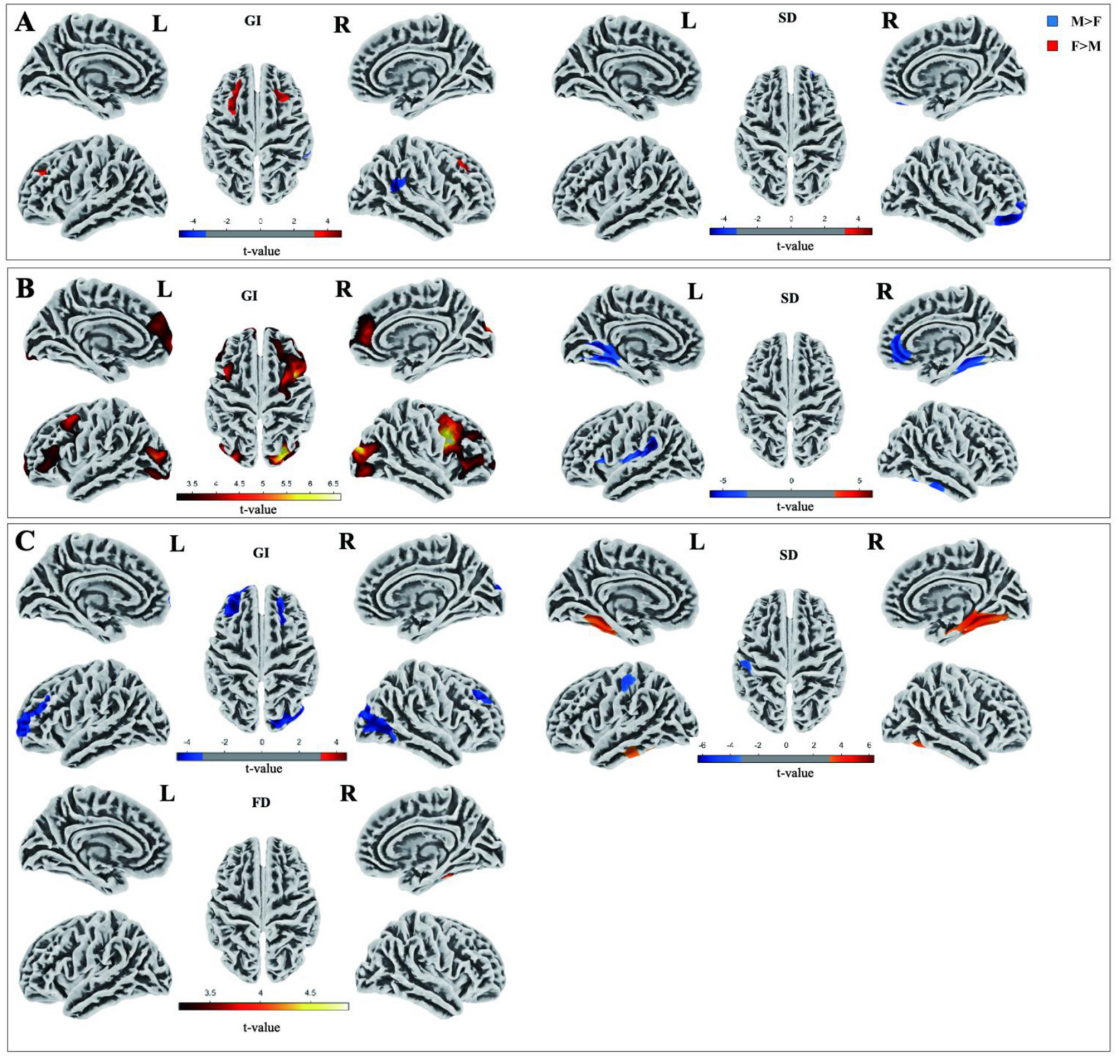
Brain regions showing intragroup differences in cortical morphological indicators between males and females in SBM analyses, **(A)** indicates differences in cortical morphometric indices between male and female healthy controls, **(B)** indicates sex differences in the PDnon-pRBD group, and **(C)** indicates sex differences in the PD-pRBD group. Negative values indicate that the indices for males were higher than those for females, and positive values indicate that the indices for males were lower than those for females (few-corrected, *P* < 0.001 for vertex, *P* < 0.05 for cluster). M, male; F, female.

SBM analysis at the vertex level revealed that male participants in the HC group exhibited higher SD indices in the right orbitofrontal cortex and lower GI indices in the bilateral rostral middle frontal region than female participants (Figure 2A). The trend of changes in the PDnon-pRBD group is similar to that in the HC group, with an increased extent of involvement. Specifically, males exhibited higher SD indices in the right orbitofrontal region, and lower GI indices in the bilateral superior frontal and right rostral middle frontal regions than females (Figure 2B). In the PD-pRBD group, females exhibited a higher SD index in the bilateral fusiform gyrus, a lower GI index in the right superior frontal, lateral occipital , and left rostral middle frontal regions, and a higher FD index in the right Fusiform gyrus than males (Figure 2C). For more detailed information on clustering, please refer to Supplementary Table S1.

### Alterations in brain network properties

**Figure 3:**
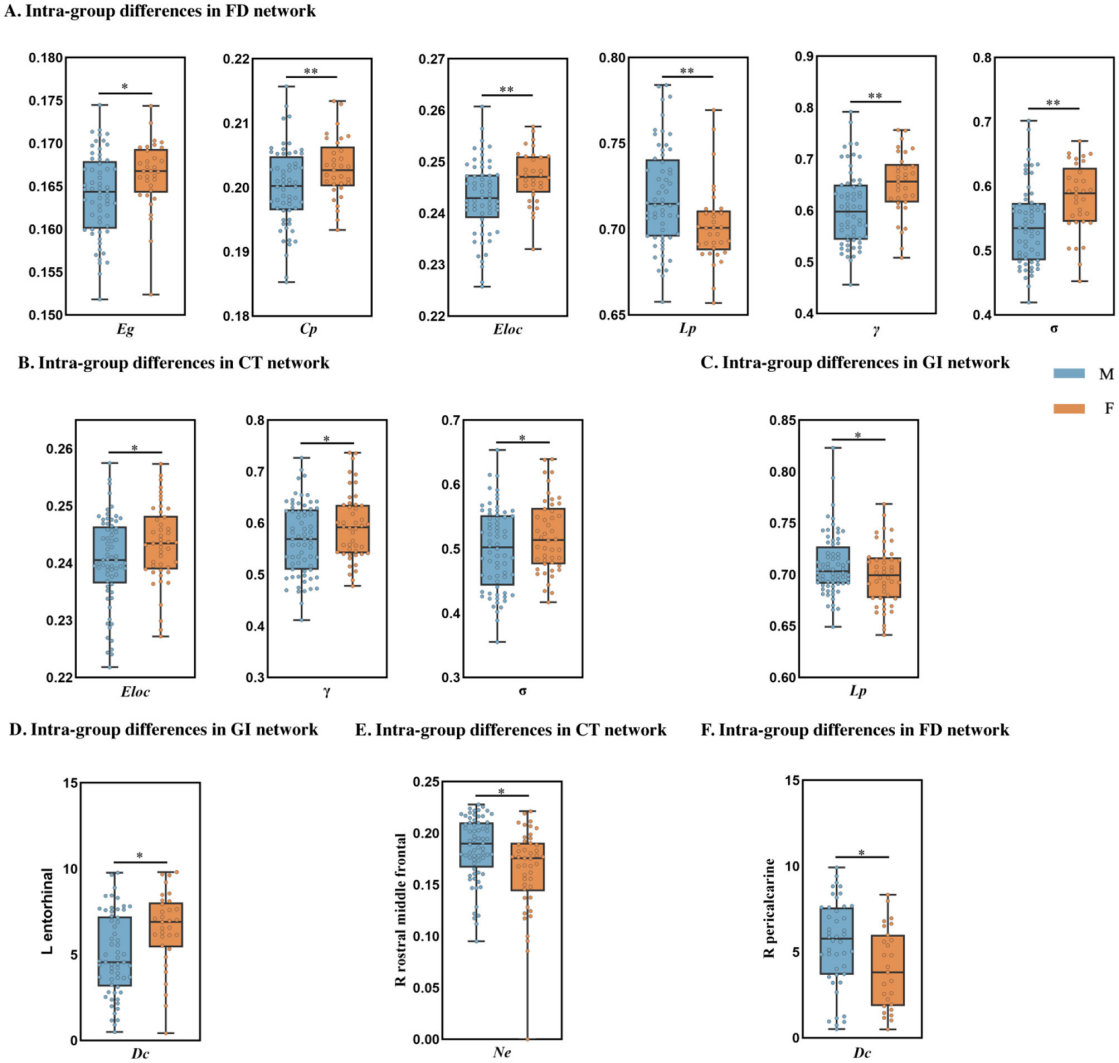
Topological characterization of the network. **(A–C)** Represent global brain network properties, **(A)** shows the findings for the PD-pRBD group, while **(B, C)** show the findings for the PDnon-pRBD group, **(D–F)** represent nodal brain network properties. **(D)** Shows the findings for the PD-pRBD group; **(E)** shows the findings for the PDnon-pRBD group, and **(F)** shows the findings for the HC group. *Denotes *P* < 0.05 and ** denotes *P* < 0.01. M, male; F, female. Eg, Global efficiency; Cp, Clustering coefficient; Eloc, Local efficiency; Lp, Characteristic path length; γ, Normalized clustering coefficient; σ, Small world; Ne, Nodal efficiency; Dc, Degree centrality; Bc, Betweenness centrality.

In the morphology-based similarity networks constructed using cortical metrics, changes in global indices between PDnon-pRBD and PD-pRBD groups are similar. In the FD morphological networks of the PD-pRBD group, the *Eg* (*P* = 0.015), σ (*P* = 0.001), and γ (*P* = 0.002) in males are lower than those in females, while the characteristic *Lp* (*P* = 0.005) is higher in males (Figure 3A). These results are confirmed in the CT and GI networks of the PDnon-pRBD group, demonstrating the robustness of the findings (Figures 3B, C). Additionally, in the PD-pRBD group, the *Eloc* (*P* = 0.003) and *Cp* (*P* = 0.007) are lower in males compared to females in the FD network (Figure 3A). No significant differences in the global topological structure were observed between males and females in the HC group.

In our study of nodal properties, we observed that male participants in the PDnon-pRBD and HC groups exhibited higher nodal attributes than their female counterparts. Specifically, in the PDnon-pRBD group, the right rostral middle frontal region of the CT network demonstrated higher *Ne* (*P* = 0.048) in males (Figure 3E). Similarly, in the HC group, the right pericalcarine region of the FD network showed higher *Dc* (*P* = 0.041) in males (Figure 3F). However, in the PD-pRBD group, males exhibited lower *Dc* (*P* = 0.020) in the left entorhinal cortex region of the GI network compared to females (Figure 3D).

### Relationships between network properties and clinical variables

**Figure 4:**
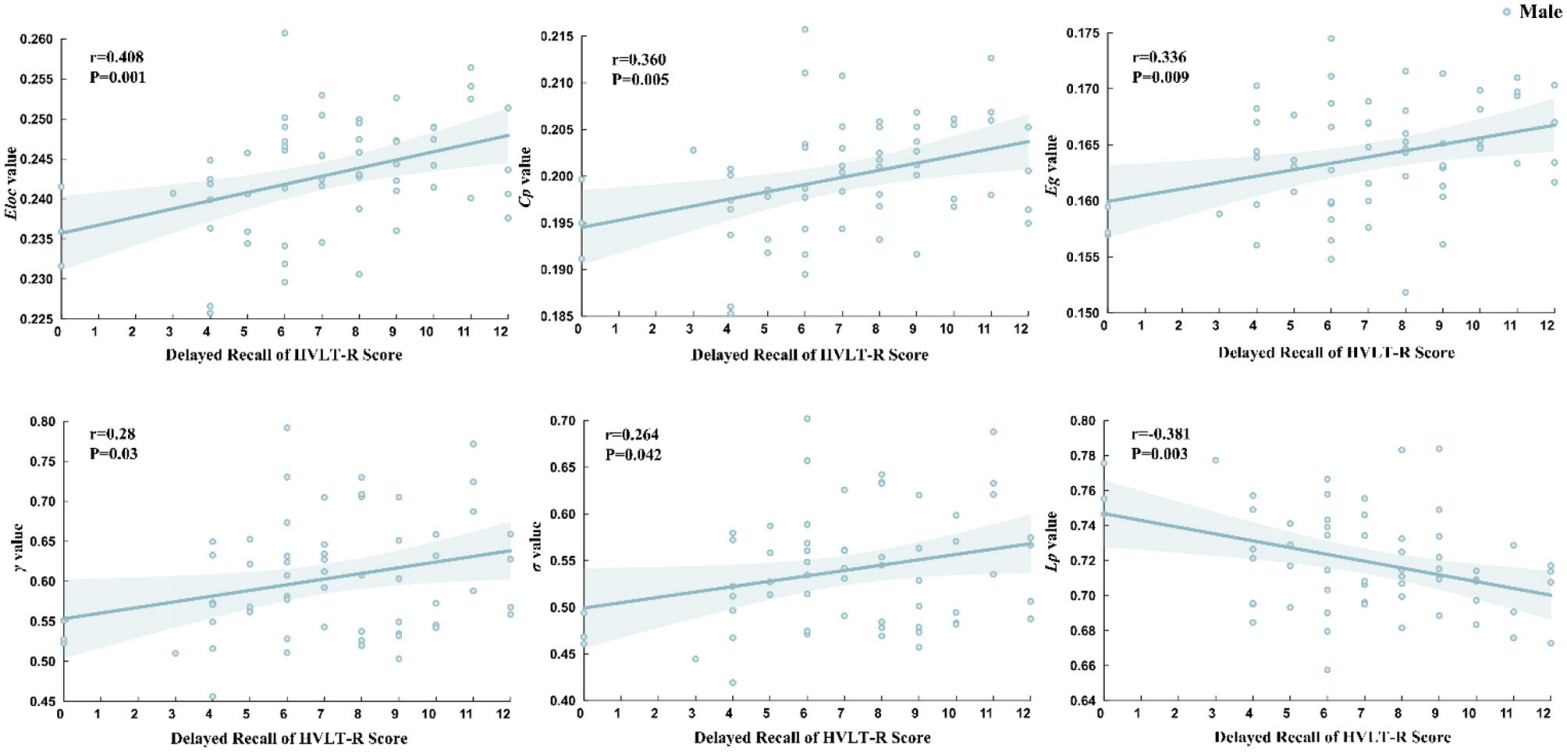
Relationships between network properties and clinical variables. The correlation between the Delayed Recall of HVLT*-*R with global brain network properties in PD-pRBD patients is illustrated in the figure. The Delayed Recall score of HVLT*-*R is positively correlated with *Eloc, Cp, Eg*, γ, and σ, and negatively correlated with the *Lp*.

Pearson correlation analysis revealed that only the global network properties in male participants from the PD-pRBD group were associated with clinical scale scores. Specifically, Delayed Recall scores from HVLT*-*R were positively correlated with *Eloc* (*r* = 0.408, *P* = 0.001), *Cp* (*r* = 0.360, *P* = 0.005), *Eg* (*r* = 0.336, *P* = 0.009), γ (*r* = 0.280, *P* = 0.03), and σ (*r* = 0.264, *P* = 0.042). Conversely, Delayed Recall scores from HVLT*-*R was negatively correlated with *Lp* (*r* = −0.381, *P* = 0.003). In contrast, no significant correlations were found between morphological networks and clinical indicators in either male or female participants from the PDnon-pRBD and HC groups (Figure 4). Additionally, no correlations were identified in females across any of the three groups.

## Discussion

This study integrates VBM, SBM, and individual morphological network analyses to explore sex-specific covariation patterns in PD patients with and without possible RBD. The results reveal progressive changes in brain structure and individual morphological network metrics from HC to PDnon-pRBD to PD-pRBD. Regarding brain structure, both males and females show consistent trends in sex differences from HC to PDnon-pRBD, whereas PD-pRBD exhibited changes opposite to those observed in the other two groups. Furthermore, in individual morphological networks, regardless of the presence of RBD, male PD patients consistently demonstrate lower brain information transmission efficiency than female patients. Notably, in the PD-pRBD group, alterations in male network topology are significantly associated with cognitive function.

Our study found that sex is closely associated with the clinical manifestations of PD-pRBD patients. The results showed that there were no significant differences in age and education level between males and females in the PD-pRBD, PDnon-pRBD, and HC groups. However, in the PD-pRBD group, significant sex differences were observed in cognitive performance: female patients generally performed better than male patients, a finding consistent with previous research (5). Specifically, compared to the other two groups, the PD-pRBD group showed greater sex differences in MoCA and delayed recall scores. This suggests that when PD patients are accompanied by RBD symptoms, females may possess certain “protective” factors in cognition, leading to better performance than males. Previous studies have also found that in later stages of PD, the decline in plasma concentrations in male patients is more pronounced than in females, and these levels are closely related to cognitive and sleep disturbances in male PD patients (45).

We systematically examined the relationships of sex with GMV and cortical morphology using VBM and SBM approaches. Research has shown that regions of the brain with reduced GMV in the PD-pRBD group play a crucial role in sleep regulation (46). This may suggest that the presence or absence of RBD symptoms is associated with differences in the degeneration patterns of various subcortical nuclei. Consequently, the study further investigates sex differences between PD patients with and without RBD symptoms. The study found that male patients with PD-pRBD exhibited lower GMV in the frontal temporal lobe than female patients. Our study also found that males demonstrated lower cognitive performance than females in the PD-pRBD group, consistent with the results of previous studies (3). The frontal and temporal lobes are known to be strongly linked to cognitive performance (47, 48). We observed that the reduction in GMV within these regions may be correlated with changes in cognitive abilities. In contrast, no significant sex-related differences in GMV were observed in the PDnon-pRBD group. Together with the results of the current study, these sex-related differences in gray matter volume in pRBD may represent a potential structural basis for the observed differences in cognitive performance among patients.

In terms of cortical morphological metrics, the study found consistent changes in the PDnon-pRBD and HC groups, with males exhibiting lower GI values in the right rostral middle frontal region compared to females. However, in the PDnon-pRBD group, this GI difference further extended to the superior frontal gyrus, indicating that the superior frontal gyrus becomes increasingly affected as PD progresses. In contrast, the changes in the PD-pRBD group were completely opposite, with males showing higher GI values in both the superior and middle frontal gyri compared to females. This trend reversal was observed in GI as well as SD values. GI reflects cortical complexity, indicating structural brain changes (49), while SD indicates the shape characteristics of the brain surface (50). The observed pattern of between-group differences in cortical complexity for patients with PD-pRBD, which contrasts with those in patients with PDnon-pRBD and HCs, indicates a shift in GI and SD for males transitioning from non-pRBD to pRBD. This may indicate that cortical complexity is particularly sensitive to the progression of degenerative diseases and may be useful for capturing trends within the neurodegenerative process (51, 52). Notably, female patients in the PD-pRBD group exhibited higher GMV in the superior frontal gyrus and middle frontal gyrus regions, although their GI was lower than that of male patients in the same group, suggesting an opposing trend. This discrepancy may arise because VBM is a composite representation of GM morphology, encompassing cortical area and cortical thickness, among other factors (53). Our study also identified altered FD values exclusively in the PD-pRBD group. In the PD-pRBD group, female patients exhibited significantly higher FD values than male patients in the Fusiform gyrus. FD, a measure of cortical complexity, condenses all cortical metrics into a single value (54) and is more reliable than individual morphological metrics. The study further revealed that alterations in FD in males were closely associated with cognitive abilities (55). This may reflect the enhanced sensitivity of FD in characterizing sex-specific structural differences and cognitive disparities (56, 57). In summary, the differences in cortical complexity between male and female patients in PD-pRBD group were more remarkable than that in PDnon-pRBD and HC groups, which further underscoring that there are possibilities of distinct trends between male and female PD patients with or without RBD.

Individual morphological networks are also crucial for investigating sex-related differences (58). In the current study, we further investigated sex-related differences in network topology attributes among PD patients with or without concomitant RBD. For global network properties, in the PDnon-pRBD and PD-pRBD groups, *Eloc*, σ, and γ were lower in male patients than in female patients, while *Lp* was higher in male patients. The differences in patients with PDnon-pRBD involved the CT and GI networks, whereas the differences in the PD-pRBD group were primarily concentrated in the FD network. *Eloc* measures a network's resilience against failures and disruptions (59); γ quantifies the average clustering coefficient, reflecting the overall connectivity between nodes; and *Lp* represents the average shortest path length between all pairs of nodes in a network (60). The observed differences in these global network metrics suggest that, irrespective of the presence of RBD, cerebral information transfer is less efficient in male patients than in female patients (41–43). Additionally, patients with pRBD exhibited more pronounced differences in global properties than patients without pRBD; specifically, male patients displayed lower *Eg* and *Cp* in the FD network than females, which complicates the integration of information across the brain (61, 62). Previous studies have demonstrated that abnormalities in global topological properties are closely linked to cognitive performance. Previous studies have shown that abnormalities in global topological properties are closely related to cognitive performance. The reduction in these regions may be closely associated with the decline in cognitive abilities in pRBD patients. Additionally, our study also reveals that the changes in the global network of male PD-pRBD patients are significantly correlated with clinical scale outcomes. It has been reported that electroencephalogram slowing and neurocognitive impairments occur in patients with RBD. The present study also shows that male patients in the RBD group exhibit more severe disease manifestations compared to their female counterparts. Integrating these findings, patients with PD-pRBD and PDnon-pRBD exhibit extensive differences in network properties, in addition to purely morphological variations, potentially representing a further network manifestation of morphological discrepancies.

Furthermore, significant sex-related differences were observed in the *Dc* and *Ne* of nodes across PD patients. These graph theoretical analysis metrics indicate the centrality of nodes within the network and the efficiency of information exchange and elucidate the brain's processes for handling information, decision-making, and task execution (63, 64). In the HC group, female participants showed lower *Dc* values in the right pericalcarine region within the FD network. In the PDnon-pRBD group, female patients showed lower Ne values in the right rostral middle frontal gyrus (MFG) within the CT network. The MFG is commonly associated with working memory (65) but may also be related to visuospatial abilities (66). This could correspond to the relatively poorer performance of female participants than male participants in visuospatial assessments in both the HC and PDnon-pRBD groups. In the PD-pRBD group, male patients exhibited lower *Dc* values in the left entorhinal cortex within the *GI* network. Pathological studies in patients with PD-pRBD have confirmed that abnormal accumulation of α-synuclein in the entorhinal cortex is a hallmark of disease progression (67). The findings of this study suggest that damage to the entorhinal cortex may have been more pronounced in male patients within the PD-pRBD group. This sex-specific difference may reflect male-specific pathological mechanisms in neurodegenerative diseases, such as variations in neuronal susceptibility or protein metabolism. The entorhinal cortex is a principal hub in the brain's memory network (68), and impaired information processing in this region may be associated with the decline in cognitive performance (69). Therefore, the observed differences in this node may be consistent with previously noted differences in *Eg* and *Cp*.

Although LEDD has been considered as a covariate in this study, there are still various treatments and factors that may influence the brain structure and function of PD patients. Therefore, it is worth further investigating the potential effects of other drug treatments. For example, dopamine agonists (such as 3,4-dihydroxy-L-phenylalanine) and monoamine oxidase type B inhibitors (such as rasagiline) may improve symptoms in PD patients, but they might also have different effects on the brain structure of these patients. Previous studies have shown that some PD patients develop motor dysfunction after long-term use of dopamine agonists (70), and this dysfunction may represent an irreversible shift from a non-motor dysfunction state to a motor dysfunction state due to drug treatment, a process that may be accompanied by structural changes in the brain. Past animal model studies have found that PD rats treated with 3,4-dihydroxy-L-phenylalanine showed increased volumes in the globus pallidus internus and substantia nigra pars reticulata after the onset of motor dysfunction (71), and similar structural changes have been verified in clinical studies (72). Additionally, functional magnetic resonance imaging studies have found significant changes in the connectivity of the right inferior frontal gyrus with the left motor cortex and right putamen in patients taking dopamine agonists after motor dysfunction onset (73). In PD animal models, long-term treatment with rasagiline has been shown to reduce dopaminergic cell loss in the substantia nigra (74, 75), while some studies also suggest that rasagiline may have chronic effects on the structure and function of the basal ganglia in PD patients (76). Therefore, these changes may provide further evidence for the relationship between drug effects and brain structural alterations.

Recent studies suggest that sex differences in neurodegenerative diseases may arise from the synergistic interaction of multiple mechanisms, including genetic regulation, sex hormones, and the vulnerability of the dopaminergic system. At the genetic level, the expression of Parkinson's disease-related genes shows significant sexual dimorphism, with certain genes in male Parkinson's patients (such as PARK6 and PARK7) being more significantly downregulated compared to females (77). This sex-specific gene dysregulation may impair the ability of male neurons to cope with pathological damage. The protective effects of sex hormones provide a key advantage for females: estrogen exerts neuroprotective effects through a dual mechanism—directly inhibiting the pathological aggregation of α-synuclein and stabilizing its fibrillar structure (77), while also enhancing microglial immune regulatory functions to reduce neuroinflammatory responses (78). The sex-specific vulnerability of the dopaminergic system is also noteworthy: dopaminergic neurons in the substantia nigra pars compacta are highly sensitive to oxidative stress and mitochondrial dysfunction, with female neurons demonstrating stronger resilience (78). In summary, the multidimensional interaction of genetic, hormonal, and neurotransmitter systems leads to distinct sex differences in the risk and progression of Parkinson's disease.

This study has certain limitations. First, the data used in this research were derived from the PPMI database, which includes data from multiple centers and scanners worldwide. While there are strict guidelines and protocols for acquiring clinical and imaging data to ensure standardization (24, 79), some degree of heterogeneity remains. In addition, there is an imbalance in the proportions of males and females in each subgroup, which may have a potential impact on the analysis results. Especially when analyzing sex differences, the smaller female group may result in certain subtle morphological differences not being fully captured. We are currently collecting RBD-related data in a clinical setting, with plans to expand the sample size and further validate and complement the findings. Second, this study did not use polysomnography (PSG) to identify RBD. Although the RBDSQ score has high sensitivity and specificity (26), it may overestimate the incidence of RBD, leading to false positives. This limitation is particularly relevant in subgroup analyses where false positives could influence the comparison of morphological and network differences between pRBD and non-pRBD groups. These false positives may obscure or exaggerate differences, and we acknowledge that PSG, as the gold standard for RBD diagnosis, should be used in future studies for more accurate classification of pRBD. Moreover, key clinical data such as treatment interventions and comorbidities were not included in this study; we plan to supplement these data in the future to improve the interpretability of the results. Lastly, this study only used T1-weighted imaging data for morphological analysis and did not incorporate multimodal MRI data to explore PD. Previous studies have shown that quantitative susceptibility mapping (QSM) has clear advantages in assessing iron deposition in PD (80–82). In the future, we plan to consider using techniques such as QSM to investigate sex differences in PD patients, with the goal of providing more precise support for clinical diagnosis.

## Conclusion

Overall, our findings indicate that patients with PD with and without concomitant RBD exhibit notable sex-specific patterns at both the morphological and network levels. These sex-related differences were more pronounced and cognitively relevant in patients with RBD than in those without RBD. These results may reflect differences in neuroplasticity between men and women during the progression of both PD and RBD. In particular, they underscore the importance of considering sex-related factors in understanding the neuropathological mechanisms of PD accompanied by RBD.

## Data Availability

The datasets presented in this study can be found in online repositories. The names of the repository/repositories and accession number(s) can be found below: Parkinson's Disease Progression Marker Initiative (PPMI) (PPMI, http://www.ppmi-info.org).
