# Supplementary material for: Sex-specific brain morphological and network differences in patients showing Parkinson's disease with and without possible rapid eye movement sleep behavior disorder
Source: Front Neurol. 2025 Apr 22;16:1561555. doi: 10.3389/fneur.2025.1561555 (PMC12053292; doi:10.3389/fneur.2025.1561555)
Supplement: Supplementary file 1 [file Table_1.docx]

**Supplemental data Table S1**

**SBM analysis, brain regions with intragroup differences in cortical morphological indicators were identified between male and female PD-pRBD patients (p < 0.05, family‐wise error correction)**

| **Features** | **Region** | **Hemisphere** | **MNI Peak Vertex**  **Coordinates** | | | **Cluster Size**  **(Voxel)** | | **Peak Value** |
| --- | --- | --- | --- | --- | --- | --- | --- | --- |
|  |  |  | **X** | **Y** | **Z** |  | | |
| FD | fusiform | R | 44 | -25 | -22 | 115 | 4.9 | |
| SD | fusiform | L | -42 | -37 | -24 | 839 | 6.3 | |
|  | postcentral* | L | -54 | -20 | 52 | 163 | 4.3 | |
|  | fusiform | R | 45 | -33 | -22 | 1398 | 5.5 | |
| GI | rostral middle frontal* | L | -33 | 45 | 20 | 622 | 4.5 | |
|  | lateral occipital* | R | 48 | -80 | 6 | 690 | 4.3 | |
|  | superior frontal* | R | 23 | 42 | 33 | 234 | 3.6 | |

Note: Atlas labeling was performed according to the Desikan–Killiany atlas.

Abbreviations: L, left; RH, right.

The symbol * represents that the number of men exceeds the number of women.

**SBM analysis, brain regions with intragroup differences in cortical morphological indicators were identified between male and female PDnonRBD patients (p < 0.05, family‐wise error correction)**

| **Features** | **Region** | **Hemisphere** | **MNI Peak Vertex**  **Coordinates** | | | **Cluster Size**  **(Voxel)** | | **Peak Value** |
| --- | --- | --- | --- | --- | --- | --- | --- | --- |
|  |  |  | **X** | **Y** | **Z** |  |  | |
| SD | insula* | L | -47 | -41 | 25 | 1213 | 5.9 | |
|  | lingual* | L | -31 | -51 | -8 | 952 | 4.8 | |
|  | fusiform* | R | 35 | -46 | -12 | 735 | 4.6 | |
|  | medial orbitofrontal* | R | 9 | 37 | -19 | 352 | 4.4 | |
| GI | caudal middle frontal | L | -42 | 13 | 51 | 459 | 4.6 | |
|  | lateral occipital | L | -36 | -80 | 9 | 435 | 5.3 | |
|  | superior frontal | L | -9 | 67 | -2 | 428 | 4.2 | |
|  | rostral middle frontal | R | 34 | 14 | 27 | 2560 | 6.6 | |
|  | lateral occipital | R | 29 | -83 | 15 | 953 | 6.1 | |
|  | superior frontal | R | 14 | 45 | 11 | 442 | 4.8 | |

Note: Atlas labeling was performed according to the Desikan–Killiany atlas.

Abbreviations: L, left; RH, right.

The symbol * represents that the number of men exceeds the number of women.

**SBM analysis, brain regions with intragroup differences in cortical morphological indicators were identified between male and female HC(p < 0.05, family‐wise error correction)**

| **Features** | **Region** | **Hemisphere** | **MNI Peak Vertex**  **Coordinates** | | | **Cluster Size**  **(Voxel)** | | **Peak Value** |
| --- | --- | --- | --- | --- | --- | --- | --- | --- |
|  |  |  | **X** | **Y** | **Z** |  | | |
| SD | lateral orbitofrontal* | R | 21 | 43 | -16 | 284 | 4.5 | |
| GI | inferior parietal | R | 47 | -47 | 18 | 270 | 4.8 | |
|  | rostral middle frontal* | R | 32 | 29 | 43 | 227 | 4.3 | |
|  | rostral middle frontal* | L | -30 | 11 | 51 | 255 | 4.2 | |

Note: Atlas labeling was performed according to the Desikan–Killiany atlas.

Abbreviations: L, left; RH, right.

The symbol * represents that the number of men exceeds the number of women.
